# Experience of Discrimination and Oral Health Self-Perception: A Cross-Sectional Study among Brazilian Adults

**DOI:** 10.3390/ijerph21060743

**Published:** 2024-06-06

**Authors:** Renato Vitor Vieira, Carlos Antonio Gomes da Cruz, Gizelton Pereira Alencar, Viviane Elisângela Gomes, Loliza Luiz Figueiredo Houri Chalub, Anna Rachel dos Santos Soares, Maria Luiza Viana Fonseca, Ichiro Kawachi, Raquel Conceição Ferreira

**Affiliations:** 1 Department of Social and Community Dentistry, School of Dentistry, Universidade Federal de Minas Gerais, Belo Horizonte 31270-901, MG, Brazil; renatovieira@ufmg.br (R.V.V.); carlosagc@ufmg.br (C.A.G.d.C.); vivianegomes@ufmg.br (V.E.G.); lcfigueiredo@ufmg.br (L.L.F.H.C.); annasoares@ufmg.br (A.R.d.S.S.); mlvfonseca@ufmg.br (M.L.V.F.); 2 Department of Epidemiology, School of Public Health, Universidade de São Paulo, São Paulo 05508-220, SP, Brazil; gizelton@usp.br; 3 Department of Social and Behavioral Sciences, Harvard T.H. Chan School of Public, Boston, MA 02115, USA; ikawachi@hsph.harvard.edu

**Keywords:** discrimination, oral health, health inequities, social determinants of health

## Abstract

This cross-sectional study investigated the association between experiences of discrimination and oral health self-perception among a probabilistic cluster sample of Brazilian adults who participated in the 2013 National Health Survey. Oral health self-perception was categorized into three groups (very good + good; fair; poor + very poor). Reported experiences of discrimination included attributions based on the respondent’s race/skin color, social class, income, occupation, illness, sexual orientation, religion, sex, and age. Covariates included sociodemographic data, oral health conditions, access to healthcare services, health habits, mental health, and participation in social and/or religious activities. Data were analyzed using ordinal logistic regression for non-proportional odds, considering sample weights and complex samples. Among 60,202 adults, 5.84% perceived their oral health as poor + very poor, with a significantly higher proportion among those experiencing discrimination (9.98%). Adults who experienced discrimination were 1.39 times more likely to report a “poor/very poor/fair” oral health self-perception compared to those who did not experience discrimination. Those who suffered discrimination were 1.28 times more likely to have a “very poor/poor” oral health self-perception than their counterparts who were not affected by discrimination. These findings underscore the importance of considering discrimination experiences as part of the social determinants influencing oral health.

## 1. Introduction

Discrimination can be understood as the process by which a member of a socially defined group is treated differently (especially unfairly) because of his/her/their membership [1]. Social groups experiencing discrimination can be determined by several characteristics, including race, skin color, ethnicity, gender, disability, sexual orientation, or another categorical status [1]. Interpersonal discrimination refers to the perceived discriminatory interaction between individuals in their institutional roles (e.g., employee and employer, healthcare professional, and service user) or as public and private individuals (e.g., seller and buyer). In all cases, discrimination occurs when individuals act unfairly towards members of groups that are defined as being socially subordinate, reinforcing a relationship of domination and subordination. This differential treatment can happen in many places, including healthcare services. Furthermore, structural discrimination occurs when resources, such as housing, safe neighborhoods, quality education, jobs, and consumer credit access, are allocated differently among groups [1].

Discrimination has been identified as a social determinant of health inequities [1,2,3]. The mechanisms to explain the effects of discrimination on health include psychosocial harms, barriers to healthcare access and social resources, and exposure to a greater risk of violence (e.g., by the police). Discrimination is a stressor that triggers affective reactions with biological dysregulation and psychosocial responses (increased risk of stress, psychological distress, anxiety, and malaise). Dealing with these emotional states can lead to maladaptive behavioral coping responses, such as smoking and drinking. The internalization of discrimination can also result in attitudes aimed at avoiding situations or environments that negatively stereotype individuals, which can generate changes in the pattern of service use [3].

The role of different structural, individual, and contextual social determinants in the distribution of clinical and subjective oral health outcomes has been consistently demonstrated, with worse conditions found in more socially disadvantaged groups [4,5,6]. Discrimination has recently been considered a factor that may explain persistent health inequities among social groups, even after considering other socioeconomic factors. Hence, there are ample grounds for assuming the role of discrimination in oral health outcomes. Discrimination is also associated with an increase in risk factors, such as obesity, smoking, and alcohol misuse, in addition to abandoning behaviors that promote good health and adherence to personal care [3,7,8], conditions that can also boost the incidence of oral diseases [9,10,11,12,13]. This aspect is aligned with the approach to common risk factors for chronic diseases, reinforcing the need for integrated strategies to improve health [14].

Previous studies have shown that perceived discrimination due to race, skin color, or ethnicity in different settings, including healthcare facilities, is associated with poorer oral health-related quality of life among older Chinese immigrants in the United States [15] and with greater oral health disability (toothache, discomfort due to the appearance of the mouth, and food limitations) among Australian adults [16]. Similarly, perceived experiences of discrimination in healthcare services were associated with worse oral health-related quality of life among older Brazilian adults [17]. Perceptions of discrimination due to ethnicity, culture, skin color, language, or religion were also invoked as part of the variation in negative oral health self-perception among immigrants in Indonesia [18].

Recognizing the role of discrimination in oral health disparities suggests several policy implications and interventions. Healthcare providers should be trained to identify and address their biases, ensuring equitable treatment for all patients. Policies promoting cultural competence and diversity in healthcare settings can reduce discrimination and improve health outcomes. Additionally, public health initiatives must address the broader social determinants of health, advocating for equitable resource distribution and supportive environments that mitigate the adverse effects of discrimination.

In the present study, we sought to investigate the association between experiences of discrimination and perceived oral health in the Brazilian context. A measure of oral health self-perception was adopted as the outcome, as it expresses the interpretation of experiences and health status in the context of daily life in a multidimensional aspect [19].

## 2. Materials and Methods

### 2.1. Study Design, Population, and Sample

This is a cross-sectional study based on data from the National Health Survey (NHS), conducted in 2013 in Brazil. The data were extracted from the website of the Brazilian Institute of Geography and Statistics (IBGE, in Portuguese) on 4 August 2022 and incorporate updates made on 25 August 2020, including the change in sample weights due to the 2018 revision of the projection of the population of the twenty-six states and the Federal District by sex and age for the period 2010–2060. All variables were described in the database dictionary [20].

The sample comprised adults 18 years or older. The sampling was stratified with three clustering stages: census sectors or sets of sectors (Primary Sampling Units—PSUs), households (Second-Stage Units), and adult residents (18 years of age or older) (Third-Stage Units). Ten to fourteen households were randomly selected in each PSU. One resident per household, aged 18 years or older, was selected with equal probability from the list of eligible residents. A detailed description of sample size calculations can be consulted in previous publications [21]. The National Commission of Ethics approved this study in Research (CONEP). All participants signed the written informed consent (CAAE: 10853812700000008).

### 2.2. Data Collection and Variables

Data were collected through interviews with residents >18 years of age by interviewers trained by the IBGE [20] from August 2013 to February 2014.

**Figure 1:**
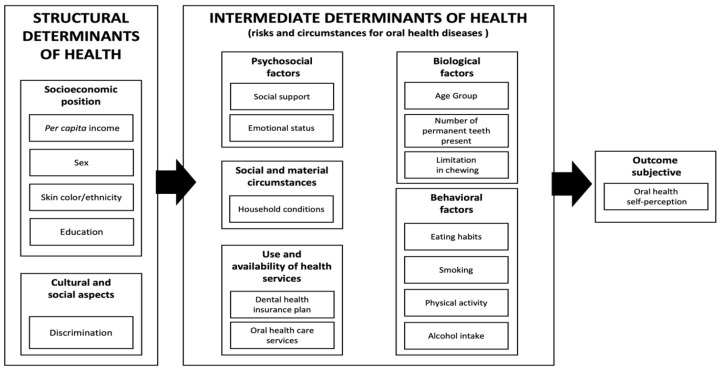
A theoretical framework to evaluate the association between experiences of discrimination and oral health self-perception based on the models of Solar and Irwin (2010) [22], Watt and Sheiham (2012) [14], and Atchison and Gift (1998) [23].

The choice of variables was guided by a theoretical framework based on the social determinants of health model [14,22] and the model of oral health self-perception according to Atchison and Gift [23] (Figure 1).

The dependent variable was oral health self-perception as determined by the following question: “In general, how do you rate your oral health?”. Response options were grouped into “very good + good”, “fair”, and “poor + very poor” due to the low frequency in the very good (9.85%) and very poor (0.92%) categories.

The independent variable of interest was responses to the Experience of Discrimination scale in healthcare, serving as a proxy for discrimination. The following question evaluated this variable: “Have you ever felt discriminated against or treated worse than others in the healthcare service by a doctor or other health professional for one of these reasons?” (yes/no). The reasons included race/skin color, social class, lack of money, occupation type, illness, sexual orientation, religion, sex, age, or other reasons.

The selected covariates followed the aforementioned theoretical framework. Proximal determinants included biological, psychosocial, and behavioral factors, the use and availability of health services, and social and material circumstances. Biological factors included the age group and objective oral health conditions. Age was categorized as 18 to 24 years, 25 to 39, 40 to 59, and ≥60 years old. Oral health conditions included the self-reported number of permanent natural teeth, a valid measure for the evaluation of tooth loss [24], as well as limitations in chewing, considering the answers to the following question: “What degree of difficulty do you have in eating because of problems with your teeth or dentures?” (none, mild, fair, intense, or very intense). Psychosocial factors included variables related to social support, which measured the frequency within the last year: “any time” (more than once a week + once a week + a few times a year + once a year) or “not once” for engagement in artistic or sports activities conducted in groups; participation in meetings of associations, community movements, academic centers or similar; involvement in voluntary work; and participation in religious activities. Respondents also answered about how often, “some days in the last week” (fewer than half the days + more than half the days + almost every day) or “no day”, they lost interest or pleasure in doing things, had problems focusing on usual activities, and felt down, depressed, or hopeless. Behavioral factors included eating habits, smoking, alcohol intake, and physical activity. The concept of a healthy diet was evaluated by grouping the consumption of types of food by cluster analysis according to Pereira et al. (2013) [25]: beans; a tomato and lettuce salad or any salad; raw or cooked vegetables, such as cabbage, carrots, chayote, eggplant, and zucchini (not counting potatoes, cassava, or yams); red meat; chicken; fish; fruit juice; fruits; soda; milk; sweets and sandwiches; snacks; or pizzas. The participant answered how many times a week (ranging from 0 to 7) they ate each type of food. The three resulting clusters were defined as healthy, balanced, and unhealthy (Supplementary Table S1). Smoking habits were assessed based on the use of tobacco products during one’s life (never smoked, ex-smoker, and smoker). The daily intake of alcoholic beverages took into account the number of drinks consumed, while binge drinking was assessed as consuming six or more drinks (for men) or five or more drinks (for women) [26]. Those who consumed alcoholic beverages less than once a month were considered in the same category as those classified as light to moderate consumption. Physical activity was evaluated by the question, “In the last three months, have you practiced any type of physical exercise or sport? (do not consider physical therapy)” (yes/no). The use of oral healthcare services was evaluated based on the time since the last dental visit (<1 year, 1 to <2, 2 to <3, ≥3 years, never used) and whether adults had a private dental health insurance plan. Social and material circumstances included the presence of indoor plumbing in the household (yes/no) and the sewage system (general or rainwater sewage system; septic tank; rudimentary cesspit; ditch, or direct sewage into the river, lake, or sea; does not have a bathroom).

We also inquired about sex (male, female), race/skin color (white, black, brown, yellow + indigenous), per capita income, and education. The per capita income was calculated based on the ratio between the sum of all types of income for each family (household income + pension income + retirement + social security + alimony + donation + amount received for rent or lease) and the number of family members. Income ranges were based on the value of the minimum wage (MW) in 2013 (R$678.00; USD 269.05): up to ½, >½ to 1, >1 to 2, >2 to 3, >3 MW. The level of education was based on current or past experience of formal education according to the Brazilian school system and the time required to complete each level of education converted into years of study (never studied, 1–4; 5–8; 9–11; ≥12 years of study).

### 2.3. Statistical Analysis

Data were subjected to descriptive analysis to obtain absolute and relative frequencies for evaluated variables and estimate the prevalence of a negative oral health self-perception and experiences of discrimination. The dependent variable has three categories with ordinal characteristics: positive (very good + good), fair, and negative (poor + very poor) oral health self-perception. For this reason, the association was investigated using an ordinal logistic regression model, making it possible to verify the changes in the association measures when the fair category was considered in the group of a positive or negative oral health self-perception. Initially, the Brant test evaluated the assumption of proportional odds for all variables [27]. A significant test for some variables indicated that the proportional odds assumption was violated; therefore, the Partial Proportional Odds (MOPP) model was chosen (Table S2). Unadjusted analyses were conducted as preliminary steps to observe the association among discrimination, covariates, and oral health self-perception. The adjustment in the model was guided by the theoretical framework, which adopted a sequential inclusion of structural and intermediate determinants. Consequently, for theoretical reasons, all covariates were retained in the final model to ensure the adjusted measure of association was accurately obtained. Changes in the strength of association (Δ Odds Ratio: ΔOR) between experiences of discrimination and negative oral health self-perception were calculated to indicate the extent to which each group of variables in the regression could potentially explain (i.e., mediate) the association. The ΔOR was obtained by the formula [(OR_model x_ − OR_model 1_)/(OR_model 1_ − 1), with the OR_model 1_ corresponding to that obtained by the model, including the most distal determinants: experience of discrimination and variables regarding the socioeconomic position. Listwise deletion was employed to handle the missing data due to the low number of missing cases. All analyses were performed considering the complex sampling design and sample weights for the selected resident with a correction of non-interview, calibrated based on the population projection. STATA v.16 Software (Stata Corporation; College Station, TX, USA) was used to conduct the analyses.

## 3. Results

The sample consisted of 60,202 participants (93.62%). The refusal rate was 2.67%, and 3.71% of residents were not found after at least two attempts. There were missing data in 4.3% of the data across all variables, specifically in race/skin color (*n* = 3) and per capita income (*n* = 11) variables. Most adults (67.50%; 95% CI: 66.78–68.21%) perceived their oral health as very good or good. Experiences of discrimination were reported by 10.62% of the sample, primarily motivated by income (5.73%; 95% CI 5.37–6.11%) and social class (5.58%; 95% CI 5.25–5.93%) (Table 1). The frequency of experiences of discrimination was higher among adults who identified as brown/black (11.54%; 95% CI 10.9–12.2%) or indigenous (16.1%; 11.8–21.4%).

The distribution of the sample regarding the variables is shown in Table 2. Most were adults from 40 to 59 years of age (34.24%), and 52.90% were female, most with 9 to 11 years of education (34.30%), with a per capita household income between ½ to 1 minimum wage, and most of them black + brown (51.09%). Most participants lived in houses with indoor plumbing, 66.58% had access to a general sewage system, and 20.89% dispose of their waste in rudimentary cesspools, ditches, or directly into the river, lake, or sea. Most had no dental health insurance plan (69.83%). Approximately half reported having visited the dentist for more than one year. Most participants were involved in volunteer work (87.89%), did not participate in meetings (83.99%), and did not engage in sports activities (73.84%). For 70.07%, religious practice was performed at any time last year. Lack of interest or pleasure in doing things and problems focusing on usual activities were reported by 22.15% and 16.60%, respectively. Feeling down, depressed, or hopeless was reported by 21.41%. Approximately one-third were ex-smokers or current smokers, and the prevalence of light-to-moderate alcohol consumption was 38.45%. An unhealthy diet was observed for 29.46%, and 68.46% reported not engaging in physical activity in the last three months. The average number of permanent natural teeth was 23.98, and 10.2% indicated some limitations in chewing.

The bivariate analysis (Table 2) showed a higher prevalence of poor oral health self-perception among those who had experiences of discrimination (9.98%) than those without these experiences (5.35%).

The chance of a negative oral health self-perception (poor/very poor/fair) among adults who had experiences of discrimination was 1.81 times that observed in the group without experiences of discrimination. The odds ratio was 1.96 in the crude model when considering only the poor categories (poor/very poor) as a negative oral health self-perception (Table S3).

The adjusted ordinal regression model (Table 3) shows that the chance of adults reporting a “poor/very poor/fair” oral health self-perception among those who had experiences of discrimination was 1.39-fold the chance of negative self-perception among adults who were not affected by experiences of discrimination. The chance of a “very poor/poor” (negative) oral health self-perception of those who suffered discrimination was 1.28-fold the chance of negative self-perception among adults who did not experience discrimination. The association measure in this last model was lower, showing that exclusion of the fair group among those with a negative self-perception resulted in a lower probability of negative self-perception as compared to the likelihood of positive self-perception, that is, the chance in the group that suffered discrimination as compared to those who did not suffer. Thus, individuals with a fair oral health self-perception also experience a higher frequency of experiences of discrimination. Table 3 shows that the variation in proportion for the variables in the group with a fair oral health self-perception was closer to that observed in the group with a negative oral health self-perception (poor/very poor). For example, concerning the smoking variable, the highest prevalence of smokers was observed in groups with poor/very poor or fair self-perception. Conversely, the highest prevalence of non-smokers was observed in the group with good or very good oral health self-perception.

Table 4 shows that the greatest decrease in the association measure occurred when the models were adjusted for psychosocial factors. The successive inclusion of additional covariates further attenuated the odds ratios, which nevertheless remained statistically significant (Table 4).

Significant associations were observed among the covariates in the socioeconomic position, material, and social circumstances, use and availability of healthcare services, and psychosocial, behavioral, and biological factors. Female individuals with a higher income and who studied >12 years had a lower chance of a negative oral health self-perception. Black and brown individuals, in turn, were more likely to have a negative oral health self-perception as compared to white adults. Unfavorable material and social circumstances (absence of indoor plumbing and sewage in a rudimentary cesspool or ditch or directly to the river, lake, or sea), not having health insurance, and less frequent use of oral healthcare services were associated with a higher chance of a negative oral health self-perception. The psychosocial factors associated with a greater chance of a negative oral health self-perception were loss of interest or pleasure in doing things; problems concentrating on usual activities; and feeling down, depressed, or hopeless. Regarding behavioral factors, a higher chance of a negative oral health self-perception was observed for adults with a balanced or unhealthy diet (compared to health), smokers and former smokers, and adults who reported not practicing physical activities. A greater chance of negative oral health self-perception was observed for those over 40 years of age and who presented any level of limitations in chewing, with the strongest association measure appearing for the most severe limitations (fair, intense, and very intense) (Table 3).

## 4. Discussion

Adults who reported experiences of discrimination had a higher frequency of negative oral health self-perception. Consistent with prior research, other biological, behavioral, and social status factors were significantly associated with the outcome, demonstrating the relevance of using the social determinants of the health model as a theoretical framework [28,29,30,31,32,33]. The negative self-perception variable was analyzed while keeping its ordinal characteristic, contributing to the understanding that those who chose to report a fair oral health self-perception presented a distribution of variables closer to respondents with a negative oral health self-perception.

This association is consistent with previous findings that demonstrated a poorer oral health-related quality of life among older Brazilians [17], poor oral health self-perception among migrants from Indonesia in South Korea [18], and greater oral health disability in Australian adults [16], among those who reported experiences of discrimination. The psychosocial mechanisms that attempt to explain the association between discrimination and health may contribute to understanding the observed association. A meta-analysis of 134 studies [3] and a systematic review of longitudinal studies [7] supported evidence that increased levels of perceived discrimination were associated with impairment in physical and mental health, including psychosocial stress and symptoms of depression and obesity, conditions also associated with poor oral health. A recent systematic review with a meta-analysis included data from 334,503 individuals, which showed that all psychiatric diagnoses were associated with an increase in dental caries [11]. Another systematic review with a meta-analysis of fourteen studies showed a positive association between depression and oral diseases, specifically dental caries, tooth loss, and edentulism [13]. These explanation mechanisms were supported by one of the major declines in the association measure between experiences of discrimination and a negative oral health self-perception observed after fitting the regression model based on psychosocial factors. This finding suggests that psychosocial effects can mediate the association between experiences of discrimination and the outcome. The percentage of participants reporting discriminatory experiences is consistent with other cross-sectional studies carried out with Brazilian populations in which similar results were found (11.5% and 10.5%) [17,34]. Different studies around the world have found similar percentages, such as in the US, in which a cohort study on self-reported discrimination and socioeconomic status found an average of 12.6% of participants reporting discriminatory experiences in three different collections [35], and in Australia, 11% in a study on racial discrimination [16].

The experience of discrimination has also been associated with a more negative psychological response to stress, with a rise in participation in unhealthy behaviors and a decline in engagement in healthy behaviors [3]. Studies have shown an increase in smoking and unhealthy eating habits in individuals who have experienced discrimination. This finding indicates that healthy behaviors are part of how the experience of discrimination can be related to health. This argument is reinforced by the findings that the evaluated behavioral factors (smoking, consumption of alcoholic beverages, diet) contributed to reducing the association measure between discrimination and negative oral health self-perception. Consolidated evidence of the association between behaviors, such as unhealthy diets, high in sugars, and smoking with oral diseases strengthens this argument [9,10,12,36,37].

Experiences of discrimination can also represent a barrier to the use of oral healthcare services. A Brazilian study with the same sample showed that the experience of discrimination by social class, low income, and type of occupation was associated with a lower prevalence of preventive services [38]. Discrimination in healthcare services has been documented in the literature and is perceived as the total denial of care, perception of lower quality care, physical and verbal abuse, and even more subtle forms, such as making certain people wait longer [39]. These experiences can alienate service users as they represent a barrier to those seeking preventive care or treatment for oral health issues. The use of healthcare services can minimize disparities in oral health, expanding access to services according to the population’s needs. Integrating questions about discrimination into routine oral health assessments and developing personalized care plans, particularly for disadvantaged populations, are approaches to enhancing clinical practice and public health policy. Collaboration with other health providers and social workers is crucial to address comprehensive patient needs, alongside offering counseling for lifestyle changes, like smoking cessation, healthy diets, and regular physical activity. Public health policies should advocate for better access to basic amenities, universal dental coverage, and strengthening anti-discrimination laws.

The experience of discrimination reported by adults in this study was more frequently motivated by income and social class. Other studies in Brazil [40,41] reported that experiences of discrimination in healthcare services were related to financial disadvantages, demonstrating that the socioeconomic condition of these individuals puts them in a socially vulnerable situation. This social disadvantage mechanism could also explain the poor health outcomes and persistent social gradient in the distribution of oral health indicators, such as caries, tooth loss, self-perception of oral health, and edentulism among socially disadvantaged social groups [4,5,6]. This study revealed that discrimination was associated with negative oral health self-perception, even after adjusting for material and social circumstances and social position variables. It also confirmed the inequities in the outcomes between social groups defined by income, education, and race/skin color, with a higher prevalence of negative oral health self-perception among the most socially disadvantaged. This result can indicate that part of these disparities could be partially explained by experiences of discrimination, taking into consideration the aforementioned mechanisms.

This study should be analyzed considering its limitations. Discrimination was assessed through self-reporting, focusing on the perception of discrimination across various factors, such as income level, social class, race/color, occupation, illness, sexual orientation, religion/belief, sex, age, and other reasons, particularly within healthcare services, but not solely by dentists. Although it considers perceived discrimination due to various personal characteristics, it is restricted to health service scenarios. The same user may feel welcomed at one health service yet have experienced discrimination in other places or real-life situations. Additionally, the measure used does not consider multiple aspects of lifelong discrimination. The challenge of measuring discrimination must be overcome by building valid tools that assess interpersonal discrimination and discriminatory institutional mechanisms. Krieger (2014) [1] also suggested that it was necessary to consider various aspects of discrimination (type, form, agency, expression, domain, level), cumulative exposure (time, intensity, frequency, duration), pathways in which discriminatory action occurs, likely ways in which individuals face and react to situations of discrimination, as well as their health consequences, and also the effects on scientific knowledge. This study used a secondary database; therefore, the operationalization of the theoretical model was restricted to the measured variables. However, the applied social determinants of the health framework allow for the control of the association of interest by biological, behavioral, and psychosocial factors, as well as the use and access to services and the socioeconomic position. In addition, it is consistent with the mechanisms that explain the association between discrimination and health outcomes. The future investigations should include variables about the political and socioeconomic context (macroeconomic policies, social and welfare policies, public policies on education, health, and social protection) to enhance or compensate for situations of social vulnerability affecting the investigated associations.

Additionally, research with a mixed methodological approach (quantitative and qualitative) may deepen the understanding of this association and the meaning of the experience of discrimination in people’s lives. Even though the data were obtained from the National Health Survey conducted in 2013, the results provide valuable insights into the association between discrimination and oral health self-perception. However, societal and demographic changes may have occurred since then. Factors, such as shifts in cultural norms, changes in healthcare policies, and evolving patterns of discrimination, could influence the relevance of the findings today. There has not been a more recent survey that includes an evaluation of experiences of discrimination and health outcomes. This survey was an important data source for revealing this association and a starting point for new investigations. Conducting new surveys to capture updated information on discrimination and its effects on oral health would provide a more accurate picture of the current situation.

## 5. Conclusions

Experience of discrimination was associated with negative oral health self-perception, indicating that it is a social determinant that should be considered in oral health studies and actions aimed at reducing inequities in the distribution of oral diseases among social groups.

## Figures and Tables

**Table 1:** Self-perceived oral health and the reasons for discriminatory experiences of adults aged 18 and over.

	*n*	% (95% CI)
Self-perception of oral health		
Very Good	34.355	57.65 (57.60; 58.39)
Good	5.217	9.85 (9.34; 10.39)
Fair	16.050	26.66 (26.02; 27.32)
Poor	3.105	4.92 (4.62; 5.24)
Very poor	619	0.92 (0.80; 1.05)
Experiences of discrimination		
Income	3839	5.73 (5.37; 6.11)
Social Class	3803	5.58 (5.25; 5.93)
Occupation	1201	1.66 (1.49; 1.85)
Other reasons	892	1.60 (1.42; 1.80)
Diseases	1065	1.57 (1.40; 1.75)
Race/color	1061	1.46 (1.30; 1.63)
Age	911	1.26 (1.10; 1.44)
Religion/belief	504	0.82 (0.69; 0.97)
Sex	241	0.38 (0.29; 0.49)
Sexual orientation	153	0.18 (0.14; 0.23)

**Table 2:** Distribution and percentage of Brazilian adults (>18 years old) according to the experience of discrimination and the investigated covariates in the total sample and by oral health self-perception. Brazil. National Health Survey. 2013.

Oral Health Self-Perception					
	Total Sample		Very Good + Good	Fair	Poor + Vary Poor
	*n*	% (95% CI)	% (95% CI)	% (95% CI)	% (95% CI)
Experiencie of discrimination					
Did not suffer discrimination	53,264	89.38 (88.87;89.87)	68.97 (68.22; 69.72)	25.68 (25.01; 26.36)	5.35 (5.01; 5.70)
Suffered discrimination of any kind	6938	10.62 (10.13; 11.13)	55.08 (53.05; 57.09)	34.94 (33.08; 36.85)	9.98 (8.82; 11.28)
Socioeconomic position					
Sex					
Male	25,920	47.10 (46.34; 47.87)	65.95 (64.90; 66.99)	28.03 (27.08; 29.00)	6.02 (5.54; 6.53)
Female	34,282	52.90 (52.13; 53.66)	68.88 (67.94; 69.80)	25.44 (24.61; 26.29)	5.68 (5.28; 6.11)
Race/skin color (*n* = 60,199)					
White	24,106	47.56 (46.75; 48.37)	73.20 (72.22; 74.16)	22.17 (21.30; 23.06)	4.63 (4.20; 5.09)
Black	5631	9.13 (8.69; 9.60)	60.82 (58.49; 63.11)	31.27 (29.03; 33.60)	7.91 (6.74; 9.27)
Brown	29,512	41.96 (41.18; 42.74)	62.60 (61.5; 63.64)	30.58 (29.60; 31.57)	6.82 (6.32; 7.36)
Yellow + indigenous	950	1.35 (1.21; 1.51)	64.10 (58.61; 69.25)	31.93 (26.91; 37.40)	3.97 (2.74; 5.72)
Per capita income in minimum wage (MW) (*n* = 60,191)					
Up to ½	14,256	20.42 (19.81; 21.04)	56.46 (55.02; 57.89)	33.98 (32.58; 35.40)	9.56 (8.83; 10.36)
½ to 1	17,504	29.32 (28.57; 30.08)	62.43 (61.14; 63.70)	30.43 (29.26; 31.62)	7.15 (6.51; 7.84)
1 to 2	15,493	28.80 (28.11; 29.50)	70.74 (69.46; 71.98)	24.91 (23.76; 26.09)	4.35 (3.83; 4.94)
2 to 3	5335	9.34 (8.90; 9.80)	75.45 (73.30; 77.47)	20.88 (18.95; 22.95)	3.67 (2.90; 4.65)
More than 3	7603	12.12 (11.42; 12.85)	84.50 (83.00; 85.90)	13.88 (12.56; 15.32)	1.61 (1.23; 2.12)
Education (in years of study)					
Never studied	4444	6.39 (6.04; 6.74)	51.72 (48.82; 54.62)	33.33 (30.76; 35.99)	14.95 (13.04; 17.08)
1 to 4	8695	15.05 (14.46; 15.65)	59.40 (57.56; 61.22)	31.97 (30.24; 33.75)	8.63 (7.77; 9.58)
5 to 8	15,239	25.09 (24.39; 25.81)	60.61 (59.20; 62.01)	31.81 (30.53; 33.11)	7.58 (6.90; 8.33)
9 to 11	20,026	34.30 (33.56; 35.04)	70.61 (69.49; 71.70)	25.74 (24.73;26.76)	3.66 (3.21; 4.17)
≥12	11,798	19.18 (18.38; 20.01)	82.55 (81.30; 83.74)	15.21 (14.12; 16.38)	2.23 (1.82; 2.74)
Material and social circumstances					
Presence of indoor plumbing					
Yes	55,184	93.93 (93.47; 94.36)	68.53 (67.77; 69.27)	26.06 (25.39; 26.74)	5.41 (5.08; 5.76)
No	5018	6.07 (5.64; 6.53)	51.59 (49.31; 53.85)	35.93 (33.59; 38.34)	12.48 (11.04; 14.09)
Sewage system					
General or rainwater sewage system	29,823	66.58 (59.46; 61.68)	71.88 (70.88; 72.85)	23.43 (22.58; 24.30)	4.69 (4.27; 5.16)
Septic tank	12,435	15.73 (14.87; 16.62)	64.78 (63.20; 66.33)	29.36 (27.88; 30.90)	5.86 (5.21; 6.58)
Rudimentary cesspit + ditch + direct sewage into the river, lake, or sea + other	15,204	20.89 (19.96; 21.84)	59.33 (57.89; 60.76)	32.72 (31.36; 34.11)	7.94 (7.26; 8.68)
Does not have a bathroom	2576	2.81 (2.56; 3.09)	49.06 (45.74; 52.38)	36.17 (32.85; 39.63)	14.77 (12.43; 17.47)
Use and availability of healthcare services					
Private dental health insurance plan					
Yes	16,368	30.17 (29.25; 31.10)	78.65 (77.48; 79.78)	18.64 (17.56; 19.76)	2.72 (2.32; 3.18)
Not	43,834	69.83 (68.90; 70.75)	62.68 (61.82; 63.53)	30.13 (29.35; 30.93)	7.19 (6.77; 7.63)
Use of oral health services					
<1 year	25,656	44.30 (43.50; 45.11)	74.28 (73.26; 75.28)	22.00 (21.09; 22.94)	3.72 (3.30; 4.19)
1 to <2 years	11,518	19.27 (18.71; 19.85)	67.48 (65.92; 69.00)	28.35 (26.91; 29.84)	4.17 (3.65; 4.75)
2 to <3 years	5655	8.87 (8.48; 9.28)	64.36 (62.31; 66.36)	30.12 (28.22; 32.08)	5.53 (4.64; 6.57)
≥3 years	15,287	24.23 (23.55; 24.91)	58.22 (56.81; 59.61)	31.97 (30.70; 33.26)	9.82 (9.04; 10.66)
Never used	2086	3.33 (3.06; 3.61)	53.27 (49.60; 56.91)	31.14 (27.78; 34.70)	15.59 (13.14; 18.40)
Psychosocial factors					
Engagement in artistic or sports activities in groups					
Any time in the last year	14,818	26.16 (25.38;26.95]	74.82 (73.58; 76.02)	21.79 (20.71; 22.90)	3.39 (2.91; 3.96)
Not once	45,388	73.84 (73.05;74.62]	64.90 (64.07; 65.73)	28.39 (27.64; 29.15)	6.71 (6.31; 7.12)
Participation in meetings of associations, community movements, academic centers or similar					
Any time in the last year	10,302	16.01 (15.43;16.62]	68.11 (66.52; 69.66)	26.78 (25.33; 28.29)	5.11 (4.47; 5.84)
Not once	49,900	83.99 (83.38;84.57)	67.38 (66.59; 68.17)	26.64 (25.92; 27.37)	5.98 (5.61; 6.37)
Involvement in volunteer work					
Any time in the last year	6915	12.11 (11.57;12.67)	71.57 (69.73; 73.35)	24.28 (22.65; 25.98)	4.15 (3.37; 5.10)
Not once	53,287	87.89 (87.33;88.43)	66.94 (66.17; 67.69)	26.99 (26.31; 27.69)	6.07 (5.72; 6.45)
Participation in religious activities					
Any time in the last year	42,149	70.07 (69.30;70.82)	67.53 (66.68; 68.37)	27.05 (26.28; 27.83)	5.42 (5.06; 5.81)
Not once	18,023	29.93 (29.18;30.70)	67.42 (66.16; 68.65)	25.76 (24.66; 26.89)	6.82 (6.21; 7.49)
Loss of interest or pleasure in doing things					
No day	46,072	77.85 (77.19;78.49)	70.19 (69.40; 70.97)	24.96 (24.26; 25.68)	4.85 (4.52; 5.19)
Some days in the past two weeks	14,130	22.15 (21.51;22.81)	58.04 (56.52; 59.53)	32.64 (31.28; 34.03)	9.33 (8.54; 10.18)
Problems focusing on usual activities					
No day	49,656	83.40 (82.84;83.95)	69.54 (68.77; 70.30)	25.48 (24.78; 26.19)	4.98 (4.66; 5.32)
Some days in the past two weeks	10,546	16.60 (16.05;17.16)	57.23 (55.53; 58.92)	32.61 (31.03; 34.23)	10.16 (9.22; 11.18)
Felt down, depressed or hopeless					
No day	46,712	78.59 (77.93;79.23)	70.21 (69.43; 70.97)	25.05 (24.35; 25.77)	4.74 (4.43; 5.07)
Some days in the past two weeks	13,490	21.41 (20.77;22.070	57.56 (56.08; 59.03)	32.57 (31.22; 33.96)	9.87 (8.95; 10.86)
Behavior factors					
Eating habits					
Healthy	19,980	34.48 (33.67; 35.29)	74.28 (73.17; 75.37)	21.80 (20.79; 22.85)	3.91 (3.46; 4.42)
Balanced	21,998	36.06 (35.28; 36.86)	62.47 (61.34; 63.58)	29.86 (28.82; 30.93)	7.67 (7.04; 8.35)
Unhealthy	18,224	29.46 (28.80; 30.13)	65.72 (64.43; 66.98)	28.43 (27.29; 29.60)	5.85 (5.30; 6.45)
Smoking habits					
Never smoked	41,215	67.86 (67.16; 68.55)	71.24 (70.42; 72.04)	24.47 (23.73; 25.23)	4.29 (3.95; 4.65)
Ex-smoker	10,258	17.49 (16.92; 18.08)	62.45 (60.78; 64.09)	30.08 (28.57; 31.63)	7.47 (6.69; 8.33)
Smoker	8729	14.65 (14.16; 15.15)	56.21 (54.49; 57.92)	32.71 (31.09; 34.37)	11.08 (10.03; 12.22)
Intake of alcoholic beverages					
Did not report consumption	37,200	59.64 (58.82; 60.45)	66.61 (75.65; 67.56)	27.06 (26.19; 27.94)	6.33 (5.92; 6.78)
Light to moderate consumption or <once a month	21,547	38.45 (37.64; 39.27)	69.20 (68.11; 70.26)	25.82 (24.87; 26.80)	4.98 (4.50; 5.52)
Risk consumption (binge drinking)	1455	1.91 (1.74; 2.10)	61.05 (56.46; 65.45)	31.30 (27.14; 35.77)	7.65 (5.57; 10.43)
Physical activity in the last three months					
Yes	17,896	31.54 (30.78; 32.31)	75.20 (74.08; 76.29)	21.78 (20.76; 22.83)	3.02 (2.62; 3.47)
No	42,306	68.46 (67.69; 69.22)	63.95 (63.08; 64.81)	28.91 (28.15; 29.69)	7.14 (6.72; 7.59)
Biological factors					
Age group (years old)					
18 to 24	20,767	31.77 (31.09; 32.47)	74.43 (72.70;76.09)	22.55 (21.01; 24.17)	3.02 (2.43; 3.75)
25 to 39	20,435	34.24 (33.59; 34.91)	71.17 (70.06; 72.25)	24.90 (23.88; 25.95)	3.93 (3.52; 4.39)
40 to 59	11,177	18.06 (17.48; 18.65)	63.57 (62.34; 64.78)	28.58 (27.51; 29.68)	7.85 (7.18; 8.57)
>60	11,177	23.98 (23.81;24.16)	62.38 (60.72; 64.01)	29.75 (28.23; 31.32)	7.87 (7.13; 8.67)
Average number of permanent natural teeth					
Mean (standard deviation)	60,202	23.98 (23.81;24.16)	24.94 (24.73; 25.16)	22.80 (22.51; 23.07)	18.33 (17.70; 8.92)
Limitations in chewing					
None	53,336	3.59 (3.34; 3.87)	72.04 (71.29; 72.77)	24.43 (23.76; 25.12)	3.53 (3.26; 3.82)
Mild	3581	1.53 (1.37; 1.71)	35.34 (32.76; 38.01)	49.19 (46.44; 51.95)	15.47 (13.59; 17.57)
Fair	2296	3.59 (03.34;03.87)	22.76 (19.85; 25.96)	49.25 (45.71; 52.80)	27.99 (25.12; 31.04)
Intense + Very intense	989	15.93 (15.37; 16.50)	18.88 (14.78; 23.78)	25.67 (21.49; 30.35)	55.45 (49.74; 61.03)

**Table 3:** Adjusted ordinal regression model of the association between the experience of discrimination and oral health self-perception adjusted for biological, behavioral, and psychosocial factors and variables related to the use and availability of healthcare services, social and material circumstances, and socioeconomic position. National Health Survey. Brazil. 2013 (*n* = 60,188).

Oral Health Self-Perception		
Variables	Very Poor + Poor + Fair	Very Poor + Poor
	OR (95% IC)	OR (95% IC)
Experience of discrimination		
Suffered discrimination of any kind	1.39 (1.26; 1.52)	1.28 (1.07; 1.54)
Socioeconomic position		
Female versus male	0.82 (0.76; 0.89)	0.90 (0.79; 1.02)
Race/skin color black versus white	1.30 (1.15; 1.47)	1.21 (0.96; 1.52)
Race/skin color brown versus white	1.25 (1.16;1.34)	1.10 (0.95; 1.27)
Race/skin color yellow/indigenous versus white	1.52 (1.16; 2.00)	0.77 (0.49; 1.19)
Per capita income ½ to 1 MW versus <1/2 MW	0.87 (0.80; 0.94)	0.87 (0.80; 0.94)
Per capita income 1 to 2 MW versus <1/2 MW	0.74 (0.67; 0.81)	0.74 (0.67; 0.81)
Per capita income 2 to 3 MW versus <1/2 MW	0.70 (0.62; 0.81)	0.70 (0.62; 0.81)
Per capita income >3 MW versus <1/2 MW	0.51 (0.44; 0.59)	0.51 (0.44; 0.59)
1 to 4 years of study versus never studied	0.92 (0.80; 1.06)	0.78 (0.63; 0.97)
5 to 8 years of study versus never studied	1.04 (0.91; 1.19)	1.03 (0.83; 1.27)
9 to 11 years of study versus never studied	1.00 (0.86; 1.16)	0.91 (0.71; 1.17)
>12 years of study versus never studied	0.70 (0.59; 0.84)	0.79 (0.58; 1.06)
Material and Social Circumstances		
Absence of indoor plumbing	1.15 (1.01; 1.30)	1.15 (1.01; 1.30)
Septic rank versus general or rainwater sewage system	1.10 (1.01; 1.20)	1.10 (1.01; 1.20)
Rudimentary cesspit + ditch + direct sewage into the river, lake, or sea + other versus general or rainwater sewage system	1.14 (1.05; 1.25)	1.14 (1.05; 1.25)
Does not have a bathroom	1.24 (1.05; 1.46)	1.24 (1.05; 1.46)
Use and availability of healthcare services		
Did not have a private dental health insurance plan	1.27 (1.16; 1.38)	1.27 (1.16; 1.38)
Used oral healthcare services 1 to <2 years versus the last year	1.24 (1.14; 1.36)	1.00 (0.82; 1.22)
Used oral healthcare services 2 to <3 years versus the last year	1.22 (1.09; 1.36)	1.10 (0.88; 1.37)
Used oral healthcare services ≥ 3 years versus the last year	1.28 (1.16; 1.40)	1.50 (1.26; 1.77)
Never used the oral health service versus used the service the last year	1.25 (1.06; 1.48)	2.07 (1.57; 2.73)
Psychosocial factors		
Engaged in artistic or sports activities in group not once in the last year versus any time in the last year	1.09 (0.99; 1.19)	1.09 (0.99; 1.19)
Did not participate in meetings of associations, community movements, academic centers, or similar in the last year versus participated any time in the last year	0.93 (0.85; 1.02)	0.93 (0.85; 1.02)
Was not involved in volunteer work in the last year versus involved any time in the last year	1.03 (0,92; 1.15)	1.01 (0.91; 1.12)
Did not participate in religious activities in the last year versus participated any time in the last year	1.00 (0.93; 1.08)	1.21 (1.00; 1.38)
Lost interest or pleasure in doing things someday versus no day in the past two weeks	1.26 (1.15; 1.38)	1.26 (1.15; 1.38)
Had trouble focusing on usual activities some day versus no day in the past two weeks	1.14 (1.04; 1.25)	1.14 (1.04; 1.25)
Felt down and depressed or hopeless some day versus no day in the past two weeks	1.22 (1.12; 1.33)	1.22 (1.12; 1.33)
Behavioral factors		
Balanced diet versus healthy diet	1.26 (1.16; 1.37)	1.32 (1.11; 1.57)
Unhealthy diet versus healthy diet	1.18 (1.09; 1.28)	1.05 (0.88; 1.25)
Ex-smoker versus never smoked	1.16 (1.06; 1.26)	1.22 (1.04; 1.44)
Smoker versus never smoked	1.35 (1.23; 1.47)	1.73 (1.47; 2.05)
Light-to-moderate consumption of alcoholic beverages versus no alcoholic beverages	1.03 (0.96; 1.12)	0.97 (0.84; 1.11)
Risk consumption of alcoholic beverages versus no alcoholic beverages	1.17 (0.95; 1.44)	1.30 (0.90; 1.88)
Does not practice physical activities	1.12 (1.02; 1.24)	1.30 (1.10; 1.54)
Biological factors		
Age group 25 to 39 years versus 18 to 24 years	1.15 (1.03; 1.28)	1.15 (1.03; 1.28)
Age group 40 to 59 years versus 18 to 24 years	1.55 (1.37; 1.75)	1.55 (1.37; 1.75)
Age group >60 years versus 18 to 24 years	1.49 (1.27; 1.75)	1.49 (1.27; 1.75)
Number of teeth present	1.00 (1.00; 1.01)	0.99 (0.99; 1.00)
Mild limitations on chewing versus no limitations	3.81 (3.35; 4.33)	3.54 (2.98; 4.20)
Regular limitations on chewing versus no limitations	6.45 (5.37; 7.75)	6.36 (5.34; 7.59)
Intense limitations + too intense to chewing versus no limitations	6.97 (5.17; 9.39)	19.0 (14.6; 24.7)

**Table 4:** Change in the odds ratio values of the association between experience of discrimination and oral health self-perception throughout regression model fitting. National Health Survey. Brazil. 2013.

	Model 1 ^a^	Model 2 ^b^	Model 3 ^c^	Model 4 ^d^	Model 5 ^e^	Model 6 ^f^
	OR (95% CI)	OR (95% CI)	OR (95% CI)	OR (95% CI)	OR (95% CI)	OR (95% CI)
Very poor + poor + fair						
Experience of discrimination						
No	1 (Reference)	1 (Reference)	1 (Reference)	1 (Reference)	1 (Reference)	1 (Reference)
Yes	1.72 (1.58; 1.88)	1.72 (1.57; 1.87)	1.72 (1.57; 1.87)	1.50 (1.37; 1.64)	1.47 (1.34; 1.61)	1.39 (1.26; 1.52)
ΔOR	-	0	0	−30.56	−34.72	−45.83
Very poor + poor						
Experience of discrimination						
No	1 (Reference)	1 (Reference)	1 (Reference)	1 (Reference)	1 (Reference)	1 (Reference)
Yes	1.86 (1.60; 2.17)	1.86 (1.59; 2.16)	1.85 (1.58; 2.16)	1.61 (1.38; 1.89)	1.56 (1.33; 1.83)	1.33 (1.12; 1.58)
ΔOR	-	0	−1.16	−29.07	−34.88	−61.62

^a^ Model 1: association between experience of discrimination and negative oral health self-perception adjusted for socioeconomic status (income per capita, sex, race/skin color, education). ^b^ Model 2: adjusted for socioeconomic status + social circumstances (presence of indoor plumbing in the house and type of sanitary sewage). ^c^ Model 3: adjusted for socioeconomic status + social circumstances + use and availability of oral healthcare services (access to private dental health insurance; time since last dental visit). ^d^ Model 4: adjusted for socioeconomic status + social circumstances + use and availability of oral healthcare services + biopsychosocial factors (engaged in artistic or sports activities in groups, participation in meetings of associations, community movements, academic centers, or similar; involvement in volunteer work; participation in religious activities; lost interest or pleasure in doing things; had trouble focusing on usual activities; feeling down, depressed, or hopeless). ^e^ Model 5: adjusted for socioeconomic status + social circumstances + use and availability of oral healthcare services + biopsychosocial factors + behavioral factors (diet, smoking, alcohol consumption, and physical activity). ^f^ Model 6: adjusted for socioeconomic status + social circumstances) + use and availability of oral healthcare services + biopsychosocial factors + behavioral factors + biological factors (age, number of natural teeth present, and limitations in chewing).

## Data Availability

The database that support the findings of this study is available at https://www.pns.icict.fiocruz.br/bases-de-dados/, accessed on 6 June 2024.

## Supplementary Materials

The following supporting information can be downloaded at: https://www.mdpi.com/article/10.3390/ijerph21060743/s1. Table S1. Weekly average consumption of each type of food according to eating habits defined by cluster analysis. ^a^ The consumption of each type of food was evaluated based on the following question: on how many days of the week do you usually eat (type of food)? (answer ranging from 0 (never or less than once a week) to 7). * The asterisk indicates a statistically significant difference in the consumption of each type of food between the groups defined by the cluster analysis—Kruskal–Wallis Test (*p* < 0.001). Table S2. Results of the Brant test of the parallel regression assumption. ^a^ A significant test statistic provides evidence that the parallel regression assumption has been violated. MS: minimum salary. Table S3. Crude analysis of the association among experience of discrimination, covariates, and a negative oral health self-perception. National Health Survey. Brazil. 2013. Table S4. Adjustment steps of the ordinal regression model of the association between the experience of discrimination and oral health self-perception. National Health Survey. 2013.
