# Supplementary material for: Experience of Discrimination and Oral Health Self-Perception: A Cross-Sectional Study among Brazilian Adults
Source: Int J Environ Res Public Health. 2024 Jun 6;21(6):743. doi: 10.3390/ijerph21060743 (PMC11203647; doi:10.3390/ijerph21060743)
Supplement: Supplementary file 1 [file ijerph-21-00743-s001.zip › ijerph-3024617-supplementary.pdf]

Supporting Information

Table S1. Weekly average consumption of each type of food according to eating habits defined by cluster analysis.

Types of foods[a]	Healthy eating Mean (SD)	Balanced eating Mean (SD)	Unhealthy eating Mean (SD)
Beans*	**5.44 (2.17)**	5.12 (2.38)	5.09 (2.31)
Salad*	**6.04 (1.60)**	2.84 (2.38)	**2.67 (2.13)**
Vegetables*	**5.23 (2.00)**	2.31 (2.07)	**2.22 (1.83)**
Red meat*	3.59 (2.10)	3.84 (2.17)	3.87 (2.07)
Chicken*	2.98 (1.70)	2.58 (1.66)	2.66 (1.61)
Fish*	1.32 (1.43)	1.13 (1.53)	1.07 (1.39)
Fruit juice*	**4.16 (2.56)**	2.10 (2.27)	2.11 (2.22)
Fruits*	**5.67 (1.87)**	2.74 (2.29)	**3.06 (2.28)**
Soda*	1.51 (1.98)	2.79 (2.53)	**2.72 (2.53)**
Sweets*	**2.40 (2.41)**	1.98 (2.11)	2.22 (2.33)
Milk*	**5.06 (2.62)**	0.76 (1.08)	6.54 (0.93)
Sandwiches. snacks or pizza	0.96 (1.74)	0.90 (1.58)	0.95 (1.67)

[a]The consumption of each type of food was evaluated by the question: On how many days of the week do you usually eat (type of food)? (Answer ranging from 0 (never or less than once a week) to 7). * The asterisk indicates a statistically significant difference in the consumption of each type of food between the groups defined by the Cluster analysis – Kruskal-Wallis Test ($p < 0.001$).

Table S2. Results of the Brant test of parallel regression assumption.

Variables	Chi 2	P > chi2[a]
Suffered discrimination of any kind	5.82	0.016
Age group 25 to 39 years	0.01	0.936
Age group 40 to 59 years	0.91	0.341
Age group >60 years	0.20	0.655
Number of permanent natural teeth	5.51	0.019
Mild limitations in chewing	0.00	0.995
Regular limitations in chewing	6.01	0.014
Intense limitations + very intense chewing	163.54	0.000
Balanced eating habits	0.26	0.609
Unhealthy eating habit	5.63	0.018
Ex-smoker	2.04	0.153
Smoker	27.36	0.000
Light to moderate consumption of alcoholic beverages	4.63	0.031
Risky consumption of alcoholic beverages	0.53	0.465
Practice of physical activities in the last three months	8.63	0.003
Participation in meetings of associations, community movements, academic centers or similar	0.84	0.360
Engagement in artistic or sports activities in groups	1.45	0.228
Involvement in volunteer work	0.97	0.326
Participation in religious activities	8.04	0.005
Loss of interest or pleasure in doing things	0.87	0.352
Problems focusing on usual activities	1.69	0.194
Felt down. depressed or hopeless	1.14	0.286
Private dental health insurance plan	0.17	0.680
Used oral health service for 1 to < 2 years	0.66	0.418
Used oral health service 2 to < 3 years	0.55	0.457
Used the health service > 3 years	18.90	0.000
Never been to the dentist	27.54	0.000
Presence of indoor plumbing	0.00	0.997
Sanitary sewage in general sewage or rainwater network	3.42	0.064
Sewage in septic tank + rudimentary cesspit + ditch + direct sewage to the river. lake or sea + other	1.58	0.209
Per capita income ½ up to 1WM	0.60	0.440
Per capita income 1 up to 2 WM	0.06	0.807
Per capita income 2 up to 3 WM	0.06	0.810
Per capita income more than 3WM	0.00	0.962
Women	2.68	0.102
Black skin color/race	0.17	0.682
Brown skin color/ race	4.91	0.027
Indigenous + Yellow skin color/ race	6.37	0.012
1 to4 years of study	2.90	0.089
5 to8 years of study	4.16	0.042
9 to11 years of study	4.45	0.035
>12 years of study	0.40	0.529

[a]A significant test statistic provides evidence that the parallel regression assumption has been violated. MS: Minimum Salary.

Table S3. Crude analysis of the association between experience of discrimination, covariates, and a negative oral health self-perception. National Health Survey. Brazil. 2013.

	ORAL HEALTH SELF PERCEPTION	
VARIABLES	**Very poor + poor + fair**<br>OR (95% IC)	**Very poor + poor**<br>OR (95% IC)
**EXPERIENCE OF DISCRIMINATION**		
Suffered discrimination of any kind	**1.96 (1.68;2.28)**	**1.81 (1.66;1.97)**
**SOCIOECONOMIC POSITION**		
Per capita income ½ to 1 MW *versus* < 1/2 MW	**0.73 (0.64; 0.83)**	**0.78 (0.72; 0.84)**
Per capita income 1 to 2 MW *versus* < 1/2 MW	**0.43 (0.37; 0.50)**	**0.54 (0.49; 0.58)**
Per capita income 2 to 3 MW versus < 1/2 MW	**0.36 (0.28; 0.47)**	**0.42 (0.37; 0.48)**
Per capita income > 3 MW *versus* < 1/2 MW	**0.16 (0.12; 0.21)**	**0.24 (0.21; 0.27)**
Female *versus* male	0.94 (0.84; 1.05)	**0.88 (0.82; 0.93)**
Race/skin color Black *versus* white	**1.77 (1.46; 2.15)**	**1.76 (1.58; 1.96)**
Race/skin color Brown *versus* white	**1.51 (1.32; 1.72)**	**1.63 (1.53; 1.74)**
Race/skin color Yellow/indigenous *versus* white	**0.85 (0.57; 1.26)**	**1.53 (1.20; 1.94)**
1 to 4 years of study *versus* never studied	**0.54 (0.44; 0.65)**	**0.73 (0.64; 0.84)**
5 to 8 years of study *versus* never studied	**0.47 (0.39; 0.56)**	**0.70 (0.62; 0.79)**
9 to 11 years of study *versus* never studied	**0.22 (0.18; 0.27)**	**0.45 (0.39; 0.51)**
> 12 years of study *versus* never studied	**0.13 (0.10; 0.17)**	**0.23 (0.20; 0.26)**
**MATERIAL AND SOCIAL CIRCUMSTANCES**	1.13 (0.94; 1.35)	**1.39 (1.28; 1.51)**
Absence of indoor plumbing	**1.51 (1.22; 1.88)**	**1.60 (1.44; 1.78)**
Septic rank *versus* general or rainwater sewage system	**2.81 (2.42; 3.28)**	**2.07 (1.91; 2.25)**
Rudimentary cesspit + ditch + direct sewage into the river. lake or sea + other *versus* General or rainwater sewage system	**4.78 (3.77; 6.06)**	**2.53 (2.17; 2.96)**
**USE AND AVAILABILITY OF HEALTHCARE SERVICES**		
Does not have a private dental health insurance plan	**2.49 (2.14; 2.91)**	**2.04 (1.85; 2.25)**
Used oral healthcare services 1 to <2 years *versus* in the last year	**1.26 (1.08; 1.48)**	**1.39 (1.28; 1.51)**
Used oral healthcare services 2 to <3 years *versus* in the last year	**1.75 (1.52; 2.01)**	**1.75 (1.62; 1.89)**
Used oral healthcare services ≥ 3 years *versus* in the last year		
Never used the oral health service *versus* used the service in the last year	**2.78 (2.34; 3.29)**	**2.19 (2.03; 2.37)**
**PSYCHOSOCIAL FACTORS**		
Engaged in artistic or sports activities in group not once in the last year *versus* Any time in the last year	**2.04 (1.74; 2.39)**	**1.74 (1.61; 1.87)**
Did not participate in meetings of associations, community movements, academic centers, or similar in the last year *versus* participated any time in the last year	**1.53 (1.30; 1.80)**	**1.51 (1.40; 1.62)**
Did not involve in volunteer work in the last year *versus* involved any time in the last year	**1.80 (1.56; 2.08)**	**1.49 (1.38; 1.61)**
Did not participate in religious activities in the last year *versus* participated any time in the last year	**2.78 (2.43; 3.18)**	**1.92 (1.79; 2.08)**
Lost interest or pleasure in doing things some day *versus* no day in the past two weeks	**0.76 (0.66; 0.88)**	**0.89 (0.82; 0.96)**
Had trouble focusing on usual activities some day *versus* no day in the past two weeks	**1.23 (0.87; 1.74)**	**1.27 (1.05; 1.55)**
Felt down and depressed. or hopeless some day *versus* no day in the past two weeks	**2.47 (2.12; 2.89)**	**1.61 (1.50; 1.73)**
**BEHAVIORAL FACTORS**		
Balanced diet *versus* Healthy diet	**1.18 (1.01; 1.37)**	1.03 (0.95; 1.12)
Unhealthy diet *versus* Healthy diet	**2.05 (1.73; 2.42)**	**1.61 (1.50; 1.73)**
Ex-smoker *versus* Never smoked	**1.49 (1.19; 1.87)**	**1.24 (1.13; 1.37)**
Smoker *versus* Never smoked	**1.28 (1.13; 1.44)**	**1.00 (0.94; 1.07)**
Light to moderate consumption of alcoholic beverages *versus* No alcoholic beverages	**2.02 (1.80; 2.26)**	**1.70 (1.59; 1.83)**
Risk consumption of alcoholic beverages *versus* No alcoholic beverages	**2.16 (1.91; 2.43)**	**1.71 (1.58; 1.84)**
Does not practice physical activities	**2.20 (1.94; 2.49)**	**1.74 (1.62; 1.86)**
**BIOLOGICAL FACTORS**		
Age group 25 to 39 years *versus* 18 to 24 years	**1.32 (1.01; 1.70)**	**1.18 (1.06; 1.31)**
Age group 40 to 59 years *versus* 18 to 24 years	**2.74 (2.15; 3.48)**	**1.67 (1.51; 1.84)**
Age group >60 years *versus* 18 to 24 years	**2.75 (2.15; 3.51)**	**1.76 (1.57; 1.97)**
Number of teeth present	**0.96 (0.95; 0.96)**	**0.98 (0.97; 0.98)**

Mild limitations on chewing *versus* no limitation	**5.00 (4.24; 5.90)**	**4.71 (4.17; 5.32)**
Regular limitations on chewing *versus* no limitation	**10.62 (8.98; 12.56)**	**8.74 (7.33; 10.43)**
Intense limitations + too intense to chewing *versus* no limitation	**34.03 (26.67; 43.41)**	**11.07 (8.25; 14.85)**

Table S4. Adjustment steps of the ordinal regression model of the association between experience of discrimination and oral health self-perception. National Health Survey. 2013.

	ORAL HEALTH SELF PERCEPTION (Very poor + poor + fair IN COMPARISON TO very good + good)					
	**Model 1**	**Model 2**	**Model 3**	**Model 4**	**Model 5**	**Model 6**
	**OR (95% CI)**	**OR (95% CI)**	**OR (95% CI)**	**OR (95% CI)**	**OR (95% CI)**	**OR (95% CI)**
**EXPERIENCE OF DISCRIMINATION**						
Did not suffer discrimination	1	1	1	1	1	1
Suffered discrimination of any kind	1.72(1.56;1.88)	1.72(1.56;1.87)	1.70(1.56;1.86)	1.49(1.36;1.63)	1.47 (1.32;1.62)	1.39 (1.26;1.55)
**SOCIOECONOMIC POSITION**						
**Sex**						
Male	1	1	1	1	1	1
Female	0.86 (0.81;0.91)	0.86 (0.81;0.91)	0.88 (0.83;0.94)	0.78 (0.73;0.84)	0.84 (0.78;0.91)	0,83 (0,77;0,89)
**Race/skin color**						
White	1	1	1	1	1	1
Black	1.39 (1.24;1.55)	1.38 (1.23;1.55)	1.35 (1.20;1.52)	1.37 (1.23;1.55)	1.35 (1.19; 1.52)	1.28 (1.14; 1.46)
Brown	1.30 (1.21;1.39)	1.26 (1.17;1.35)	1.23 (1.15;1.32)	1.24 (1.16;1.34)	**1.25 (1.16; 1.35)**	**1.27 (1.18; 1.38)**
Yellow + indigenous	1.49 (1.16;1.92)	1.50 (1.16;1.93)	1.52 (1.18;1.96)	1.54 (1.19;1.98)	1.45 (1.12; 1.89)	1.43 (1.08;1.90)
***Per capita* income in Minimum Wage (MW) (n=60,191)**						
Up to ½	1	1	1	1	**1**	**1**
½ to 1	0.82 (0.76;0.88)	0.85 (0.74;0.98)	0.86 (0.80;0.94)	0.87 (0.80;0.94)	0.87 (0.79; 0.95)	0.84 (0.77; 0.92)
1 to 2	0.63 (0.57;0.68)	0.75 (0.67;0.85)	0.71 (0.65;0.78)	0.73 (0.66;0.80)	0.75 (0.68; 0.83)	0.73 (0.65; 0.81)
2 to 3	0.58 (0.51;0.66)	0.56 (0.49;0.64)	0.71 (0.62;0.82)	0.74 (0.64;0.84)	0.80 (0.69; 0.92)	0.73(0.63; 0.85)
More than 3	0.39 (0.34;0.45)	0.37 (0.32;0.44)	0.51 (0.44;0.58	0.52 (0.45;0.60)	0.56 (0.48; 0.66)	0.52 (0.44; 0.62)
**Education (in years of study) (n=54,547)**						
Never studied	1	1	1	1	1	1
1 to 4	0.82 (0.72;0.94)	0.85 (0.74;0.98)	0.87 (0.76;1.01)	0.87 (0.76;1.01)	0.88 (0.76; 1.03)	0.93 (0.79; 1.08)
5 to 8	0.71 (0.62;0.80)	0.75 (0.66;0.85)	0.80 (0.70;0.91)	0.83 (0.72;0.95)	0.83 (0.72; 0.96)	1.03 (0.89; 1.20)
9 to 11	0.51 (0.45;0.58)	0.56 (0.49;0.64)	0.63 (0.55;0.73)	0.68 (0.59;0.74)	0.73 (0.63; 0.86)	1.01 (0.86; 1.20)
≥ 12	0.34 (0.30;0.41)	0.37 (0.32;0.44)	0.45 (0.38;0.53)	0.48 (0.41;0.57)	0.53 (0.45; 0.64)	0.72 (0.59; 0.88)
**MATERIAL AND SOCIAL CIRCUMSTANCES**						
**Presence of indoor plumbing**						
Yes		1	1	1	1	1
No		1.20 (1.06;1.23)	1.17 (1.04;1.33)	1.19 (1.05;1.35)	1.23 (1.07; 1.42)	1.20 (1.03; 1.40)
**Sewage system**						
General or rainwater sewage system		1	1	1	1	1
Septic tank		1.14 (1.05;1.23)	1.10 (1.01;1.20)	1.10 (1.01;1.190)	1.07 (0.99; 1.17)	1.08 (0.99; 1.18)
Rudimentary cesspit + ditch + direct sewage into the river. lake or sea + other		1.22 (1.12;1.32)	1.17 (1.08;1.28)	1.16 (1.07;1.27)	1.14 (1.04; 1.24)	1.14 (1.04; 1.24)
**USE AND AVAILABILITY OF HEALTHCARE SERVICES**						
**Private dental health insurance plan**						
Yes			1	1	1	1
Not			1.28(1.17;1.39)	1.27(1.16;1.38)	1.25 (1.14; 1.38)	1.27 (1.16; 1.40)
**Use of oral health services**						
< 1 year			1	1	1	1
1 to < 2 years			1.22(1.12;1.34)	1.23(1.13;1.35)	1.20 (1.09; 1.31)	1.23 (1.12; 1.36)
2 to < 3 years			1.25(1.11;1.39)	1.25(1.12;1.40)	1.22 (1.09; 1.37)	1.23 (1.09; 1.39)
≥ 3 years			1.38(1.27;1.51)	1.37(1.25;1.49)	1.36 (1.24; 1.49)	1.32 (1.20; 1.46)
**Never used**			1.34(1.14;1.59)	1.37(1.15;1.62)	1.45 (1.20; 1.75)	1.41 (1.16; 1.71)
**PSYCHOSOCIAL FACTORS**						
**Engagement in artistic or sports activities in groups**						

Any time in the last year				1	1	1
Not once				1.28 (1.19;1.39)	1.14 (1.03; 1.26)	1.10 (0.99; 1.22)
**Participation in meetings of associations, community movements, academic centers or similar**						
Any time in the last year				1	1	1
Not once				0.89 (0.82;0.98)	0.89 (0.81; 0.98)	0.91 (0.82; 1.01)
**Involvement in volunteer work**						
Any time in the last year				1	1	1
Not once				0.97 (0.88;1.08)	0.99 (0.88; 1.10)	1.03 (0,92; 1.15)
**Participation in religious activities**						
Any time in the last year				1	1	1
Not once				1.01 (0.94; 1.09)	1.00 (0.92; 1.08)	1,02 (0.94; 1.11)
**Loss of interest or pleasure in doing things**						
No day				1	1	1
Some days in the past two weeks				1.31 (1.20;1.44)	1.23 (1.12;1.35)	1.19 (1.07; 1,30)
**Problems focusing on usual activities**						
No day				1	1	1
Some days in the past two weeks				1,26 (1.15; 1.37)	1.31 (1.19; 1.44)	1,18 (1.08; 1.30)
**Felt down and depressed or hopeless**						
No day				**1**	1	1
Some days in the past two weeks				1,37 (1,26; 1,49)	1.31 (1.20; 1.44)	1.20 (1.10; 1.32)
**BEHAVIOR FACTORS**						
**Diet**						
Healthy					1	1
Balanced					1.20 (1.11; 1.31)	1.25 (1.14; 1.36)
Unhealthy					1.12 (1.03; 1.22)	1.14 (1.04; 1.25)
**Smoking habits**						
Never smoked					1	1
Ex-smoker					1.27 (1.15; 1.38)	1.18 (1.07; 1.30)
Smoker					1.41 (1.28; 1.56)	1.32 (1.20; 1.47)
**Intake of alcoholic beverages (n= 51,790)**						
Did not report consumption					1	1
Light to moderate consumption					0.99 (0.90; 1.08)	1.03 (0.94; 1.13)
Risk consumption					1.05 (0.85; 1.30)	1,20 (0.97; 1.49)
**Physical activity in the last three months**						
Yes					1	1
No					1.19 (1.08; 1.31)	1.12(1.02; 1.24)
**BIOLOGICAL FACTORS**						
**Age group (years old)**						
18 to 24						1
25 to 39						1.10 (0,98;1.24)
40 to 59						1.54 (1.36;1.76)
>_60						1.49 (1.25;1.78)
**Average number of permanent natural teeth**						1.01 (1.00;1.01)
**Limitations in chewing**						
None						1
Mild						3.74 (3.25; 4.29)
Fair						6.11 (5.01;7.45)
Intense + Very intense						6.78 (4.88;9.41)

ORAL HEALTH SELF PERCEPTION
(VERY POOR + POOR IN COMPARISON TO VERY GOOD + GOOD + FAIR))

VARIABLES	Model 1 OR (95% CI)	Model 2 OR (95% CI)	Model 3 OR (95% CI)	Model 4 OR (95% CI)	Model 5 OR (95% CI)	Model 6 OR (95% CI)
**EXPERIENCE OF DISCRIMINATION**						
Did not suffer discrimination	1	1	1	1	1	1
Suffered discrimination of any kind	1.87(1.60;2.17)	1.86 (1.58;2.18)	1.84 (1.56;2.17)	1.61 (1.36;1.90)	1.47 (1.32;1.62)	1.28 (1.07;1.54)

**SOCIOECONOMIC POSITION**						
**Sex**						
Male	1	1	1	1	1	1
Female	0.86 (0.80;0.91)	0.86 (0.81;0.91)	0.88 (0.81;0.91)	0.78 (0.73;0.84)	0.84 (0.78;0.91)	0,83 (0,77;0,90)
**Race/skin color**						
White	1	1	1	1	1	1
Black	1.35 (1.10;1.66)	1.28 (1.04;1.57)	1.21 (0.98;1.50)	1.24 (1.00;1.53)	1.22 (0.98; 1.50)	1.14 (0.90; 1.45)
Brown	1.17 (1.02;1.34)	1.11 (0.96;1.28)	1.07 0.93;1.23)	1.08 0.94;1.25)	**1.12 (0.97; 1.29)**	**1.14 (0.98; 1.32)**
Yellow + indigenous	0.81 (0.54;1.22)	0.80 (0.53;1.22)	0.79 (0.53;1.20)	0.80 (0.53;1.21)	0.69 (0.44; 1.09)	0.64 (0.40;1.04)
***Per capita* income in Minimum Wage (MW) (n=60,191)**						
Up to ½	1	1	1	1	**1**	**1**
½ to 1	0.82 (0.75;0.88)	0.85 (0.79;0.92)	0.86 (0.80;0.94)	0.87 (0.80;0.94)	0.87 (0.79; 0.95)	0.84 (0.77; 0.92)
1 to 2	0.63 (0.47;0.68)	0.67 (0.61;0.74)	0.71 (0.65;0.78)	0.73 (0.66;0.80)	0.75 (0.68; 0.83)	0.73 (0.65; 0.81)
2 to 3	0.58 (0.51;0.66)	0.63 (0.56;0.73)	0.71 (0.62;0.82)	0.74 (0.65;0.84)	0.80 (0.69; 0.92)	0.73(0.63; 0.85)
More than 3	0.39 (0.34;0.45)	0.43 (0.37;0.49)	0.51 (0.44;0.58)	0.52 (0.454;0.60)	0.56 (0.48; 0.66)	0.52 (0.44; 0.62)
**Education (in years of study) (n=54,547)**						
Never studied	1	1	1	1	1	1
1 to 4	0.59 (0.58;0.92)	0.61 (0.49;0.75)	0.67 (0.54;0.83)	0.68(0.55;0.84)	0.64 (0.51; 0.80)	0.69 (0.55; 0.88)
5 to 8	0.47 (0.77;1.23)	0.49 (0.40;0.60)	0.60 (0.49;0.73)	0.62 (0.50;0.76)	0.57 (0.46; 0.70	0.92 (0.73; 1.17)
9 to 11	0.25 (0.68;1.18)	0.27 (0.22;0.34)	0.37 (0.29;0.46)	0.40 (0.32;0.51)	0.41 (0.32; 0.52)	0.82 (0.63; 1.08)
≥ 12	0.20 (0.53;1.02)	0.21 (0.17;0.28)	0.31 (0.24;0.41)	0.33 (0.254;0.44)	0.34 (0.26; 0.46)	0.68 (0.49; 0.94)
**MATERIAL AND SOCIAL CIRCUMSTANCES**						
**Presence of indoor plumbing**						
Yes		1	1	1	1	1
No		1.20(1.06;1.36)	1.17(1.04;1.33)	1.19(1.05;1.35)	1.23 (1.07; 1.42)	1.20 (1.03; 1.40)
**Sewage system**						
General or rainwater sewage system		1	1	1	1	1
Septic tank		1.14 (1.05;1.23)	1.10 (1.01;1.20)	1.10 (1.01;1.19)	1.07 (0.99; 1.17)	1.08 (0.99; 1.18)
Rudimentary cesspit + ditch + direct sewage into the river. lake or sea + other		1.22 (1.12;1.32)	1.17 (1.08;1.28)	1.16 (1.07;1.27)	1.14 (1.04; 1.24)	1.14 (1.04; 1.24)
**USE AND AVAILABILITY OF HEALTHCARE SERVICES**						
**Private dental health insurance plan**						
Yes			1	1	1	1
Not			1.28 (1.17;1.39)	1.27 (1.16;1.38)	1.25 (1.14; 1.38)	1.28 (1.16; 1.40)
**Use of oral health services**						
< 1 year			1 (1.1)	1 (1.1)	1	1
1 to < 2 years			0.98(0.81;1.18)	0.98(0.81;1.18)	1.20 (1.09; 1.31)	1.23 (1.12; 1.34)
2 to < 3 years			1.21(0.97;1.51)	1.19(0.95;1.49)	1.22 (1.09; 1.37)	1.23 (1.09; 1.39)
≥ 3 years			1.78(1.51;2.10)	1.73(1.47;2.04)	1.36 (1.24; 1.49)	1.32 (1.20; 1.46)
**Never used**			2.25(1.71;2.97)	2.21(1.66;2.93)	1.45 (1.20; 1.76)	1.41 (1.16; 1.71)
**PSYCHOSOCIAL FACTORS**						
**Engagement in artistic or sports activities in groups**						
Any time in the last year				1	1	1
Not once				1.28 (1.19;1.39)	1.14 (1.03; 1.26)	1.10 (0.99; 1.22)
**Participation in meetings of associations, community movements, academic centers or similar**						
Any time in the last year				1	1	1
Not once				0.89 (0.82;0.98)	0.89 (0.81; 0.98)	0.91 (0.82; 1.01)
**Involvement in volunteer work**						
Any time in the last year				1	1	1
Not once				0.98 (0.88;1.08)	1.00 (0.92; 1.08)	1.03 (0,92; 1.15)
**Participation in religious activities**						
Any time in the last year				1	1	1
Not once				1.22 (1.07; 1.38)	1.00 (0.92; 1.08)	1,02 (0.94; 1.11)
**Loss of interest or pleasure in doing things**						
No day				1	1	1
Some days in the past two weeks				1.31 (1.20;1.44)	1.23 (1.12;1.35)	1.19 (1.08; 1,30)

**Problems focusing on usual activities**						
No day				1	1	1
Some days in the past two weeks				1,26 (1.15; 1.37)	1.31 (1.19; 1.44)	1,19 (1.08; 1.31)
**Felt down and depressed or hopeless**						
No day				**1**	1	1
Some days in the past two weeks				1,37 (1,36; 1,90)	1.31 (1.20; 1.44)	1.20 (1.10; 1.32)
BEHAVIOR FACTORS						
**Diet**						
Healthy					1	1
Balanced					1.30 (1.09; 1.55)	1.32 (1.10; 1.59)
Unhealthy					1.05 (0.87; 1.25)	1.03 (0.85; 1.24)
**Smoking habits**						
Never smoked					1	1
Ex-smoker					1.48 (1.26; 1.75)	1.28(1.10; 1.52)
Smoker					2.03 (1.73; 2.39)	1.79 (1.49; 2.15)
**Intake of alcoholic beverages* (n= 51,790)**						
Did not report consumption					1	1
Light to moderate consumption					0.81 (0.69; 0.95)	0.92 (0.78; 1.09)
Risk consumption					0.99 (0,69; 1.42)	1,41 (0.97; 2.06)
**Physical activity in the last three months**						
Yes					1	1
No					1,48 (1.23; 1.78)	1.32 (1.09; 1.60)
BIOLOGICAL FACTORS						
**Age group (years old)**						
18 to 24						1
25 to 39						1.10 (0,98;1.24)
40 to 59						1.55 (1.36;1.76)
>_60						1.49 (1.25;1.78)
**Average number of permanent natural teeth**						0.99 (0.99;1.00)
**Limitations in chewing**						1
None						3.58 (2.95; 4.33)
Mild						6.45 (5.30;7.84)
Fair						18.53 (13.91;24.68)
Intense + Very intense						
N	60188	57612	57612	57612	49606	49609
